# Variation in Dietary Salt Intake Induces Coordinated Dynamics of Monocyte Subsets and Monocyte-Platelet Aggregates in Humans: Implications in End Organ Inflammation

**DOI:** 10.1371/journal.pone.0060332

**Published:** 2013-04-04

**Authors:** Xin Zhou, Ling Zhang, Wen-Jie Ji, Fei Yuan, Zhao-Zeng Guo, Bo Pang, Tao Luo, Xing Liu, Wen-Cheng Zhang, Tie-Min Jiang, Zhuoli Zhang, Yu-Ming Li

**Affiliations:** 1 Institute of Cardiovascular Disease and Heart Center, Pingjin Hospital, Logistics University of Chinese People’s Armed Police Forces, Tianjin, China; 2 Departments of Respiratory and Critical Care Medicine, Pingjin Hospital, Logistics University of Chinese People’s Armed Police Forces, Tianjin, China; 3 MRI Department, Pingjin Hospital, Logistics University of Chinese People’s Armed Police Forces, Tianjin, China; 4 Department of Physiology and Pathophysiology, Logistics University of Chinese People’s Armed Police Forces, Tianjin, China; 5 Department of Radiology, Northwestern University, Chicago, Illinois, United States of America; University Heart Center Freiburg, Germany

## Abstract

**Background:**

Monocyte activation and tissue infiltration are quantitatively associated with high-salt intake induced target organ inflammation. We hypothesized that high-salt challenge would induce the expansion of CD14++CD16+ monocytes, one of the three monocyte subsets with a pro-inflammatory phenotype, that is associated with target organ inflammation in humans.

**Methodology/Principal Findings:**

A dietary intervention study was performed in 20 healthy volunteers, starting with a 3-day usual diet and followed with a 7-day high-salt diet (≥15 g NaCl/day), and a 7-day low-salt diet (≤5 g NaCl/day). The amounts of three monocyte subsets (“classical” CD14++CD16-, “intermediate” CD14++CD16+ and “non-classical” CD14+CD16++) and their associations with monocyte-platelet aggregates (MPAs) were measured by flow cytometry. Blood oxygen level-dependent magnetic resonance imaging (BOLD-MRI) was used to evaluate renal hypoxia. Switching to a high-salt diet resulted in CD14++ monocyte activation and a rapid expansion of CD14++CD16+ subset and MPAs, with a reciprocal decrease in the percentages of CD14++CD16- and CD14+CD16++ subsets. In vitro study using purified CD14++ monocytes revealed that elevation in extracellular [Na^+^] could lead to CD14++CD16+ expansion via a ROS dependent manner. In addition, high-salt intake was associated with progressive hypoxia in the renal medulla (increased R2* signal) and enhanced urinary monocyte chemoattractant protein-1 (MCP-1) excretion, indicating a temporal and spatial correlation between CD14++CD16+ subset and renal inflammation. The above changes could be completely reversed by a low-salt diet, whereas blood pressure levels remained unchanged during dietary intervention.

**Conclusions/Significance:**

The present work demonstrates that short-term increases in dietary salt intake could induce the expansion of CD14++CD16+ monocytes, as well as an elevation of MPAs, which might be the underlying cellular basis of high-salt induced end organ inflammation and potential thromboembolic risk. In addition, this process seems largely unrelated to changes in blood pressure levels. This finding provides novel links between dietary salt intake, innate immunity and end organ inflammation.

## Introduction

Emerging evidence suggests that the immune system plays an important role in high-salt intake induced target organ injury [1], [2]. The impact of monocyte/macrophage infiltration on end organ inflammation has been demonstrated for many years [3], [4]. Monocytes are a population with functional heterogeneity. Currently, three monocyte subsets with functional heterogeneity could be differentiated: classical CD14++CD16-, intermediate CD14++CD16+, and non-classical CD14+CD16++ monocytes [5]. Recent studies indicate that monocyte subset dynamics is not only an important pathophysiological entity, but also have prognostic values for adverse cardiovascular events [6]–[11]. So far, the impact of dietary salt intake on monocyte subset homeostasis remains unclear.

High-salt intake has been previously shown to increase platelet reactivity [12]. Data in support of this conclusion, however, are derived from studies using *in vitro* methodology, and *in vivo* evidence is limited. Monocyte-platelet aggregates (MPAs) are a sensitive marker for platelet activation and play an important role in thrombotic disorders [13]–[15]. It remains unclear if high-salt intake would have an impact on MPA formation.

Chronic hypoxia is proposed as a common pathway leading to renal dysfunction with diverse etiologies and is closely associated with the onset and progression of hypertension [16]. It is estimated that more than 90% of renal oxygen consumption is used for tubular sodium transport via Na^+^/K^+^-ATPase [17], which renders the kidney more susceptible to hypoxia during increased salt intake. This will ultimately lead to increased monocyte/macrophage infiltration into the interstitium and may further exacerbate renal function [18]. In addition, increased fluid shear stress was shown to promote renal endothelial and monocyte activation [19], [20]. Because high-salt intake is associated with enhanced urinary flow rates and, therefore, presumably increased tubular fluid shear stress, it is conceivable that high-salt intake may also induce monocyte recruitment via an intra-renal hydrodynamic force dependent mechanism [21].

Therefore, the present work was designed to determine: 1) the relationship between variation in dietary salt intake and monocyte subsets and monocyte contributions to MPAs; 2) the association between high dietary salt intake-induced changes in monocyte subpopulations and end organ inflammation, focusing on functional dynamics in the kidney revealed by blood oxygen level-dependent magnetic resonance imaging (BOLD-MRI). The current work would provide a novel pathophysiological link between dietary salt intake, innate immunity and end organ inflammation.

## Methods

### Eligibility and Recruitment

Healthy non-smoking volunteers were recruited by advertisement. The exclusion criteria included cardiovascular disease (stroke, heart failure, myocardial infarction and peripheral artery disease), diabetes mellitus, hematological disorders, cancer, current stage-2 and -3 hypertension (SBP≥160 mmHg and/or DBP≥100 mmHg), secondary hypertension, abnormal routine urinary test, previous or current abnormal renal function, and symptoms of upper respiratory tract infection and/or body temperature ≥37.5 degrees Celsius during investigation. All participants provided written informed consent. The protocol was approved by the ethical committee of Pingjin Hospital, which was in accordance with the Declaration of Helsinki.

### Study Protocol

**Figure 1:**
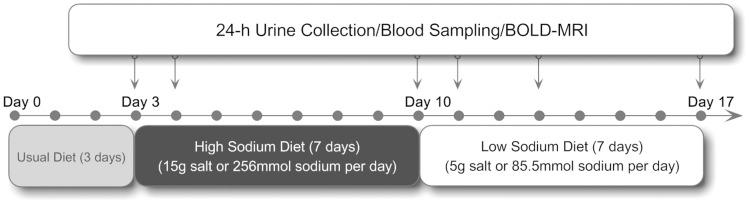
Research protocol.

We conducted a three-phase dietary intervention study including usual-salt, high-salt and low-salt feeding. Our recent investigation in aged inhabitants in rural northern China demonstrated an average of 240 mmol Na^+^/day (∼14 g salt) in 24 h urinary samples [22]. Considering sodium loss during sweating, we thus chose 15 g salt (256 mmol Na^+^)/day as high-salt loading. The low-salt level is based on World Health Organization’s recommendations (5 g salt or 85.5 mmol Na^+^/day). During the first 3 days, participants were required to eat all their meals in the hospital’s cafeteria. From day 4 to day 17, all foods were prepared by study dietitians and provided by the investigators. The participants were required to eat their meals at a defined place while being monitored by the investigators. Dietary components used to construct low- and high-salt diets are shown in **Table 1**. A detailed research protocol is shown in **Figure 1**.

**Table 1:** Dietary Components Used to Construct Low and High Salt Diets.

Dietary Component	Low Salt Diet (g/d)	High Salt Diet (g/d)
Cereals	350	350
Legumes	25	25
Vegetables	500	500
Fruits	300	300
Meats	100	100
Milk	200	200
Eggs	50	50
Fish	50	50
Oils	25	25
Salt	5	15

Two diets provide identical calorie amount (∼2300 kcal/day), and were composed of 58% carbohydrate, 15% protein and 27% fat.

### Blood Pressure Measurement and 24 h Urine Collection

All blood pressure and heart rate measurements were performed by the same investigator between 8∶00 am and 10∶00 am and recorded by an automatic Omron HEM-7200 device (Omron Inc., Dalian, China) in a sitting position after 10 min of rest. Participants were asked to provide six 24 h urine samples in a 4000 mL wide-neck plastic container (days 3, 4, 10, 11, 13 and 17). Measurements included urinary volume and concentrations of sodium, potassium and creatinine. Glomerular filtration rate (GFR) was estimated using creatinine clearance (CCr) and was calculated as follows: CCr = (urinary creatinine×urinary volume)/serum creatinine.

### Flow Cytometry Analysis of Monocyte Subsets and MPAs

**Figure 2:**
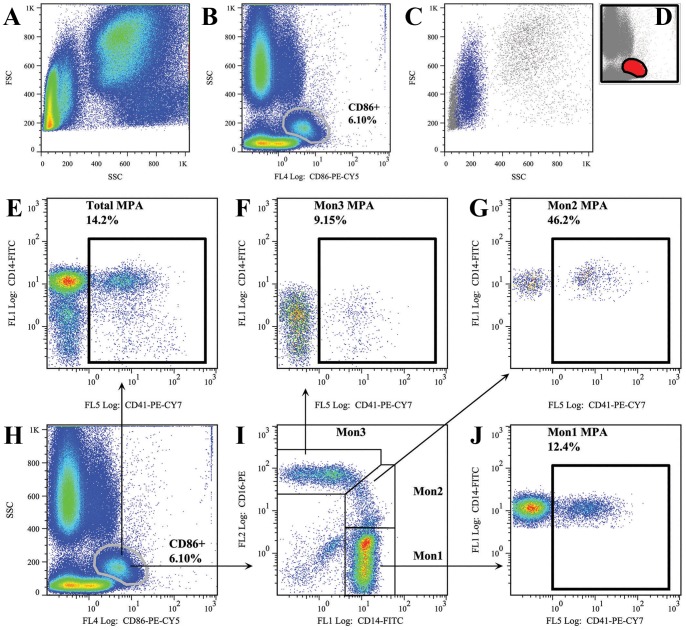
Gating strategies for monocyte subset and MPA analysis. Figures A to D show the verification analysis (backgating). Figure A shows the FSC/SSC plot for white blood cells after red blood cell lysis. Figure B shows the gating of CD86+ cells. Figure C and D shows the backgating for plot B. The CD86+ population was backgated into the FSC/SSC plot; all events fall within the traditional “monocyte gate”. Figures E to J show the detailed gating strategies for monocyte subsets and their associations with MPAs. Mon1 indicates CD14++CD16− monocytes; Mon2 indicates CD14++CD16+ monocytes; Mon3 indicates CD14+CD16++ monocytes; MPA indicates monocyte-platelet aggregates.

Flow cytometry analysis of circulating monocyte subsets and MPAs was performed according to a modified version of previously published protocols based on CD86 gating strategies [6], [8], [23], [24]. Briefly, a 21-G needle and a light tourniquet were used for blood sampling via the antecubital vein, and the first 10 mL of blood was used for biochemical assays and monocyte isolation. Blood for flow cytometry analysis was gently transferred to sodium citrate anticoagulated tubes. 50 µL whole blood was incubated with an antibody mixture containing 10 µL FITC-labeled anti-human CD14 (clone M5E2), 10 µL PE-labeled anti-human CD16 (clone 3G8), 10 µL PE-Cy5-labeled anti-human CD86 (clone IT2.2) and 10 µL PE-Cy7 labeled anti-human CD41 (clone HIP8) for 15 min at room temperature and kept protected from light. Then, 1 mL red blood cell lysis buffer was added and the solution was incubated for 10 min. The following isotype controls were used: IgG2a-FITC (clone MOPC-173), IgG1-PE (clone MOPC-27), IgG2b-PE-Cy5 (clone MPC-11), and IgG1-PE-Cy7 (clone MPOC-21), which were obtained from BioLegend (San Diego, CA, USA). Unstained, single stained and Fluorescence Minus One (FMO) controls were used for setting compensation and gating boundaries. 50 µL Flow-Count™ fluorescent microbeads (Beckman-Coulter, Miami, FL, USA) were added for absolute counting. Data were acquired using a Cytomics FC500 cytometer (Beckman-Coulter, Miami, FL, USA) and analyzed using FlowJo software (Treestar, Ashland, OR, USA). At least 150, 000 events were collected in each sample. To avoid a time dependent increase of MPAs after blood sampling, [25] flow cytometry analysis was performed within one hour after blood was drawn. Detailed gating strategies for monocyte subsets and MPAs are shown in **Figure 2**. The laboratory coefficient of variation for absolute monocyte count by flow cytometry is 2.0%, and for surface markers is less than 5.0%.

### Monocyte Purification, Intracellular Reactive Oxygen Species (ROS) and Gene Expression Analysis

Monocytes from blood samples of the participants were harvested on day 3, 4, 10, 11 and 17. Briefly, Ficoll density gradient centrifugation (Histopaque®-1077, Sigma-Aldrich, St Louis, MO, USA) was used to isolate peripheral blood mononuclear cells (PBMCs). PBMCs were further purified by positive selection with anti-CD14 magnetic microbeads (Mitlenyi Biotec, Bergisch Gladbach, Germany) in an optimized PBMC to CD14 microbead ratio as suggested previously [26]. The purity of isolated monocytes was analyzed by flow cytometry.

Intracellular ROS in freshly purified CD14++ monocytes was detected by 2′, 7′-dichlorofluorescein diacetate (DCFH-DA, Sigma Aldrich, St Louis, MO, USA). Tubes containing 2×10^4^ cells and 10 nM DCFH-DA in 1 mL DMEM were incubated at 37 degrees Celsius for 30 min in the dark. DCFH-DA was cleaved by intracellular esterase to form non fluorescent 2′, 7′- dichlorodihydrofluorescein (DCFH), which was finally converted to the highly fluorescent compound 2′, 7′-dichlorofluorescein (DCF) by ROS. DCF fluorescence was immediately detected by the FC500 flow cytometer. Ten thousand events were collected for each sample. The intracellular ROS levels within the monocyte were determined by median fluorescence intensity (MFI).

Samples collected on days 3, 10 and 17 were used for total RNA extraction and gene expression analysis. Total RNA of CD14++ monocytes was isolated using Trizol (Invitrogen, Carlsbad, CA, USA) according to the manufacturer’s protocol. Four subjects’ RNA samples were excluded from analysis because of poor quality determined by total denatured RNA electrophoresis. The remaining 48 samples from 16 subjects were used for analysis. Total RNA was then reverse transcribed by incubating with Oligo-(dT) primers (Roche, Basel, Switzerland) and M-MLV reverse transcriptase (Promega, Madison, WI, USA) according to the manufacturer’s instructions. Real-Time PCR was performed using Power® SYBR green Master Mix (Roche, Switzerland) on an ABI Prism 7300 system (Applied Biosystems, Foster City, CA, USA). The PCR primers are shown in **Table 2**. Relative expression levels were normalized with GAPDH and calculated with the 2^−ΔΔCT^ method [27].

**Table 2:** PCR Primers and Expected Amplification Products.

Gene	Sequence(5′–3′)	Length (bp)	Tm (°C)
NF-κB	Forward: CTAACACCAGCGTTTGAGGG	175	58.2
	Reverse: CTATTAAGGCACTTGAGAAGAGGG		59.1
MCP-1	Forward: CCCTTCTGTGCCTGCTGCTC	172	61.7
	Reverse: GCTTCTTTGGGACACTTGCTG		52.4
RANTES	Forward: ACCCGAAAGAACCGCCAAGT	135	62.8
	Reverse: GAGCAAGCAGAAACAGGCAAAT		60.8
TGF-β	Forward: AGCAACAATTCCTGGCGATAC	137	58.1
	Reverse: CTAAGGCGAAAGCCCTCAAT		57.7
Arg1	Forward: GGGGAAGACACCAGAAGAAGTAA	80	58.1
	Reverse: CCCGAGCAAGTCCGAAACAA		62.0
GAPDH	Forward: AGCCACATCGCTCAGACAC	66	55.3
	Reverse: GCCCAATACGACCAAATCC		55.9

NF-κB indicates nuclear factor kappa B; MCP-1 indicates monocyte chemoattractant protein-1; RANTES indicates Regulated on Activation, Normal T Cell Expressed and Secreted (*or* CC chemokine ligand 5, CCL5); TGF-β indicates transforming growth factor β; Arg1: arginase 1; GAPDH indicates glyceraldehyde-3-phosphate dehydrogenase.

### In Vitro Salt Challenge Experiments

To exam the effect of excessive salt intake on monocyte subset homeostasis, circulating monocytes were purified by CD14 magnetic beads from healthy donors on usual diet, and were incubated in serum free RPMI-1640 medium with or without additional NaCl (25 mM, which yields a hypertonic state equaling 350 mOsm/L, compared RPMI-1640 medium alone or the plasma, which is of ∼300 mOsm/L). N-acetylcysteine (NAC, 2 mM) was used as ROS scavenger. Cells were gently shacked to avoid adherence during incubation. After 2 to 6 hours, cells were harvested for the determination of intracellular free Na^+^, ROS production, and monocyte subset phenotyping (CD14 and CD16 expression). Intracellular free Na^+^ concentration was detected using the dye CoroNa™ Green (C36676, Invitrogen; in vitro *K_d_* 80 mM, excitation 488 nm, emission at 516 nm), and the fluorescence signal was detected by a FC500 flow cytometer.

### Renal BOLD-MRI

A total of 11 participants finished serial magnetic resonance imaging on days 3, 4, 10, 11 and 17 using a Phillips Intera 3.0 Tesla whole body magnetic resonance system (Philips Medical Systems, Netherlands) with a six-channel flexible matrix coil to receive the MR signal, according to previously described methods [28]–[30]. Coronal and axial T2*-weighted images were acquired in order to covering two kidneys. BOLD-MRI T2*-weighted images were recorded during a single breath holding and with a respiratory-triggered FFE sequence. Twenty slices with a thickness of 5 mm and a gap of 0 mm were obtained in the coronal plane through the kidney. The scanning sequence had the following parameters: repetition time = 120 ms; echo time from 10 ms to 48 ms; step time = 0.2 ms; flip angle = 45°; matrix size = 132×109 and field of view = 400×372.

BOLD-MRI images were analyzed based on a modified protocol from previously published work [31]. We selected 8 images in which the cortex and medulla anatomic boundaries were clear from twenty T2* weighted images and the R2* maps were calculated using ImageJ (NIH, Bethesda, MD, USA) on a pixel-by-pixel basis by fitting the corresponding echo time. Regions of interest (ROI) with unfixed size (60–90 pixels) were defined at the upper, middle and lower poles of both kidneys in the medulla and cortex based on the anatomical images. Six total ROIs (and twelve total for both kidneys) were placed in the medulla and cortex of one kidney (3 in the cortex and 3 in the medulla); each ROI excluded big vessels and renal sinus. ROIs were selected in the medulla and the cortex by the same experienced investigator and measured independently.

### Biochemical Assays

Plasma renin activity and serum levels of angiotensin II and aldosterone were determined by radioimmunoassay. Other serum biochemical assays included serum electrolytes, total cholesterol (TC), high-density lipoprotein (HDL-C), triglyceride (TG), creatinine (and urinary creatinine) and blood urea nitrogen (BUN). Urinary sodium and potassium levels were determined by atomic absorbance spectrophotometry.

### Enzyme-Linked Immunosorbent Assay (ELISA)

Urinary 8-hydroxy-2-deoxyguanosine (8-OHdG, Abcam Inc., USA, cat. no. AB101245) and serum and urinary monocyte chemoattractant protein-1 (MCP-1/CCL2, R&D, USA, cat. no. D400) were measured using commercially available ELISA kits. To exclude contamination of red and white blood cells and proteins due to menstruation, MCP-1/CCL2 were determined only in urinary samples from male subjects (n = 10).

### Statistical Analysis

Sample size estimation was based on a pilot study that measured the mean difference of CD14++CD16+ counts between baseline (day 3) and one day after high-salt loading (day 4). This difference, found to 5 cells/µL with a standard deviation of 5 cells/µL, led to an estimated sample size of 14 to provide 95% power in a two-tailed approach to detect a difference of CD14++CD16+ counts between baseline and one day after high-salt loading. To compensate for dropout, 20 subjects were planned to be recruited. The Shapiro-Wilk test was used to assess normality of quantitative variables. Continuous variables with normal distributions are presented as the mean ± SEM; otherwise they are expressed as median with interquartile range. To test the differences across time, a one-way repeated-measures ANOVA with Newman-Keuls post hoc analysis was used. If the data failed normality tests, a Friedman test followed by a Dunn’s test for multiple comparisons were performed. Correlations between two continuous variables were calculated using Pearson’s or Spearman’s correlation coefficient. All statistical analysis was performed using GraphPad Prism version 5 (GraphPad Prism Software Inc., San Diego, CA, USA). A two-tailed *P* value <0.05 was considered statistically significant.

## Results

### Blood and Urinary Biochemical Changes

A total of 36 volunteers were enrolled. They provided their 24 h urinary and blood samples at baseline. Twenty-two subjects were agreed to participate in the dietary intervention study. During high-salt intervention, 2 subjects withdrew due to intolerance to a high-salt diet or increased premature ventricular beats. The remaining 20 participants that finished the study had the following characteristics: 10 men and 10 women, mean age of 29.75±1.95 years, and mean body mass index of 21.92±0.69 kg/m^2^.

The time-dependent changes of 24 h urinary parameters are shown in **Table 3**. The salt intake, determined by 24-h urinary sodium, had mean values of 9.4 g (161.2 mmol), 18.0 g (308.1 mmol) to 18.8 g (324.3 mmol), and 3.7 g (63.3 mmol) to 4.6 g (78.6 mmol), at baseline, high-salt phase, and low-salt phase, respectively. Glomerular filtration rate (GFR), as estimated by CCr, was significantly increased in the high-salt phase and returned to baseline levels in the low-salt phase (**Table 3**). There was no obvious change in blood pressure, whereas heart rate was significantly increased during the high-salt period (*P* = 0.0007) and regressed to baseline level during the low-salt intervention (**Table 4**). Serum/plasma biochemical alterations are shown in **Table 5**. In general, lipid profiles remained stable, despite a slight decrease of high density lipoprotein cholesterol. There was a statistically suppressed serum aldosterone level during the high-salt period, while the dynamics of plasma renin activity and angiotensin II did not reach statistical significance.

**Table 3:** 24-h Urinary Parameters and Creatinine Clearance during Dietary Intervention (n = 20).

	Day 3 (baseline)	Day 4 (high-salt)	Day 10 (high-salt)	Day 13 (low-salt)	Day 17 (low-salt)	*P* for trend
UV (mL)	1400±189	2280±159	2106±140	1393±170	1454±106	<0.0001
UNa (mmol/24 h)	161.2±12.4	308.1±25.2	324.3±11.8	63.3±5.9	78.6±6.4	<0.0001
UK (mmol/24 h)	30.5±2.8	37.3±2.7	37.2±2.3	25.3±1.7	27.7±2.1	<0.0001
UNa/UK	5.66±0.46	8.37±0.36	7.93±0.41	2.53±0.18	2.90±0.14	<0.0001
UCr (mmol/24 h)	6.78±0.51	9.99±1.10	8.68±0.71	6.54±0.71	6.17±0.50	<0.001
CCr (mL/min)	89.9±5.39	120.5±10.9	124.7±7.44	94.7±6.65	90.5±3.97	<0.0001

UV indicates urinary volume; UNa indicates urinary sodium; UK indicates urinary potassium; UCr indicates urinary creatinine; CCr indicates creatinine clearance.

**Table 4:** Blood Pressure and Heart Rate Changes during Dietary Intervention (n = 20).

	baseline	Day 4 (high-salt)	Day 10 (high-salt)	Day 11 (low-salt)	Day 17 (low-salt)	*P* for trend
SBP (mmHg)	117.8±3.1	115.9±3.2	120.1±3.4	114.2±2.9	118.5±2.8	0.6677
DBP (mmHg)	65.0±2.3	62.0±2.0	69.4±2.2	66.0±1.8	68.1±2.1	0.4915
MAP (mmHg)	85.6±2.4	81.4±2.1	86.3±2.5	83.0±2.0	84.9±2.1	0.6718
HR (bpm)	72.5±1.7	81.5±2.1	80.6±2.1	76.7±2.9	75.5±2.6	0.0007

SBP indicates systolic blood pressure; DBP indicates diastolic blood pressure; MAP indicates mean blood pressure; HR indicates heart rate.

**Table 5:** Serum/Plasma Biochemical Changes During Dietary Intervention (n = 20).

	baseline	day 10 (high-salt)	day 17 (low-salt)	*P* for trend
CHO (mmol/L)	3.93±0.12	3.91±0.13	3.80±0.13	0.2716
TG (mmol/L)	0.98±0.11	1.10±0.12	1.07±0.11	0.2975
LDL-C (mmol/L)	2.52±0.04	2.48±0.04	2.43±0.04	0.3481
HDL-C (mmol/L)	1.16±0.16	1.12±0.18	1.08±0.17	0.0203
BUN (mmol/L)	4.51±0.19	4.31±0.26	4.28±0.19	0.5324
Na^+^ (mmol/L)	138.6±0.68	139.0±0.69	133.5±0.67	<0.0001
K^+^ (mmol/L)	4.52±0.10	4.18±0.07	4.00±0.13	0.0187
PRA (ng/mL/h)	2.26±0.40	1.98±0.35	2.44±0.39	0.5315
AngII (pg/mL)	41.02±5.94	48.82±2.58	60.49±3.92	0.1100
Aldosterone (pg/mL)	95.53±4.21	83.15±5.98	91.16±4.58	0.0436

CHO indicates cholesterol; TG indicates triglyceride; LDL-C indicates low density lipoprotein cholesterol; HDL-C indicates high density lipoprotein cholesterol; BUN indicates blood urea nitrogen; PRA indicates plasma renin activity; AngII indicates angiotensin II.

### Monocyte Subsets and Monocyte-Platelet Aggregates (MPAs)

We pooled baseline data from 36 participants to determine the cross-sectional relationship between spot 24 h urinary sodium and monocyte subset counts/percentages. However, no correlation was observed (for CD14++CD16+ percentages, Spearman *r* = 0.0630, *P*>0.05).

**Figure 3:**
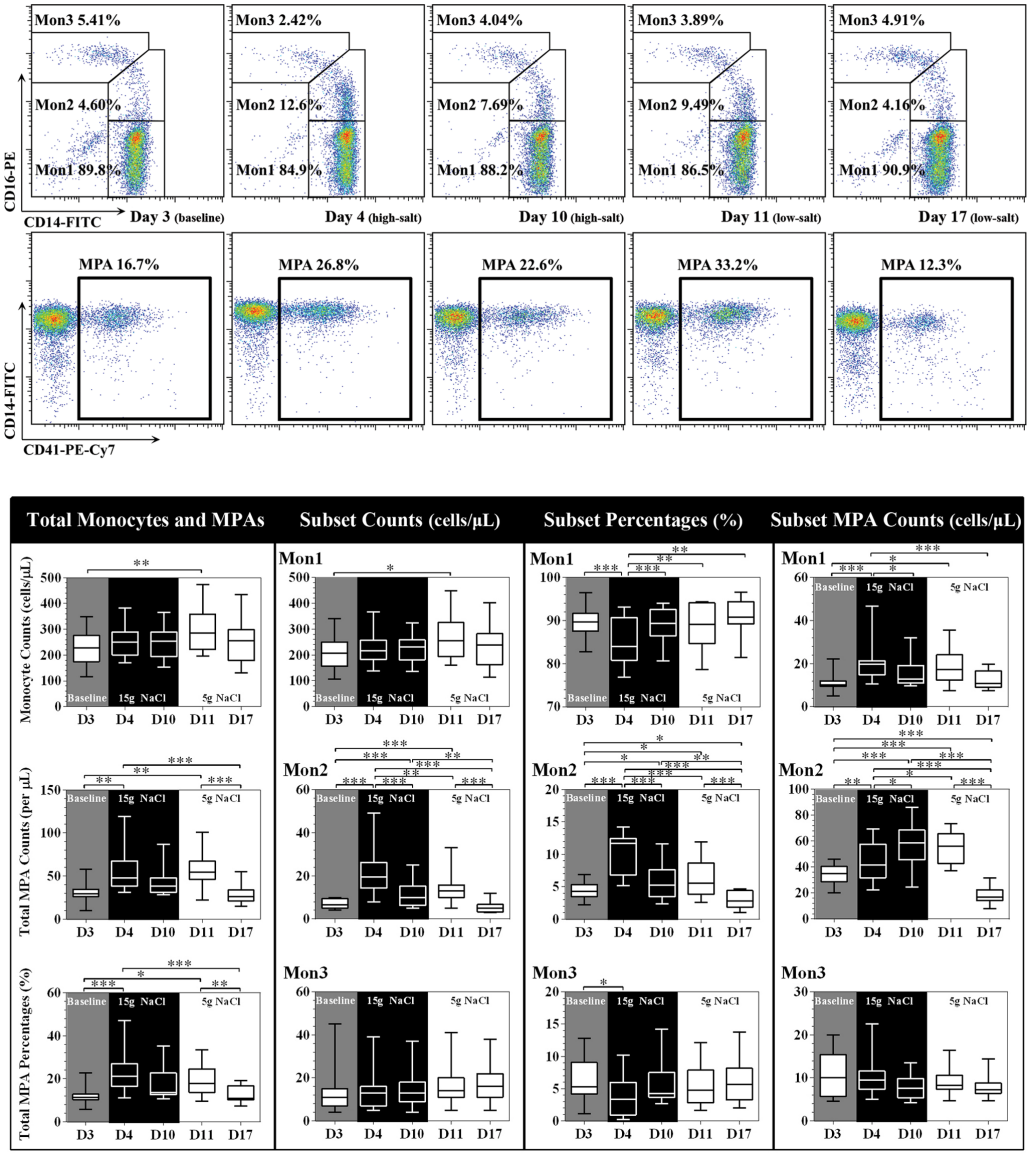
Representative flow cytometry analysis of monocyte subsets and MPAs from one participant and related statistical comparisons. Mon1 indicates CD14++CD16− monocytes; Mon2 indicates CD14++CD16+ monocytes; Mon3 indicates CD14+CD16++ monocytes. The box and whisker plots: the boxes extend from the 25^th^ to the 75^th^ percentile, with a line at the median. The whiskers extend above and below the box to show the 5^th^–95^th^ percentiles of values. “D3” to “D17” labeled in the x-axis indicates “day 3″ to “day 17″. *P<0.05, **P<0.01, ***P<0.001 (one way repeated measures ANOVA or Friedman test, n = 20).

Monocyte subset dynamics are shown in **Figure 3**. Total monocyte count remained constant despite a slight increase on day 11 (low-salt), with a concomitant increase of CD14++CD16− and CD14++CD16+ subsets. CD14++CD16+ counts/percentages increased on day 4 (high-salt), which was accompanied by a reciprocal decrease in CD14++CD16− and CD14+CD16++ percentages. This relationship tended to be normalized when the high-salt diet persisted. On day 17, the expansion of the CD14++CD16+ pool was completely regressed.

The MPA dynamics are shown in **Figure 3** **.** With regard to total MPA count and percentage, we observed a significant increase on day 4, followed by a slightly decreasing trend up to day 11. In addition, the increased MPA level was normalized and comparable with baseline by day 17. Similar patterns were observed in CD14++CD16− MPAs. However, CD14++CD16+ MPAs showed unique dynamics, undergoing a progressive increase during high-salt feeding and reaching a plateau on day 11, before abruptly decreasing up to day 17. They were statistically different compared to other time points (all *P*<0.001). Interestingly, no detectable changes had been observed in CD14+CD16++ MPAs.

**Figure 4:**
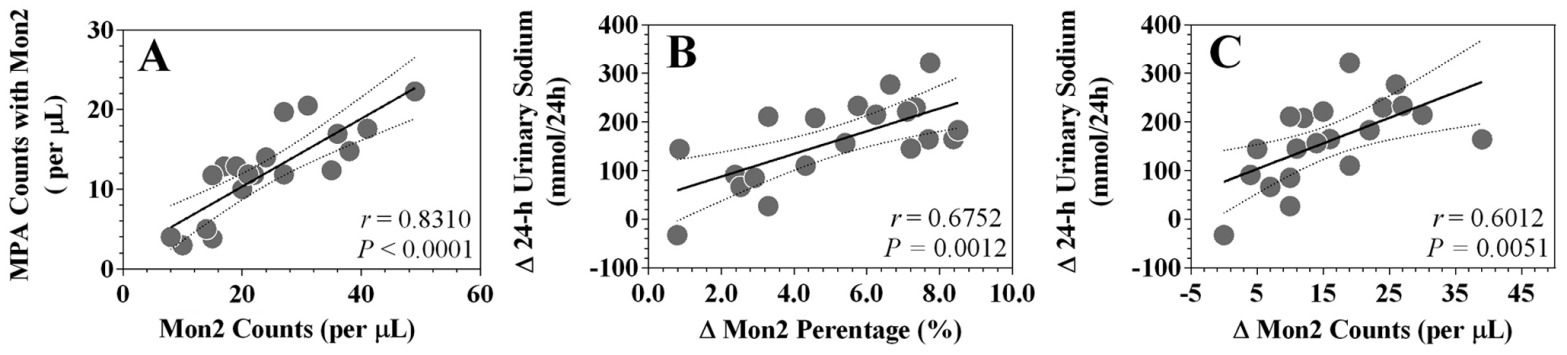
CD14++CD16+ monocyte linear correlation analysis. A, shows the correlation between CD14++CD16+ counts and CD14++CD16+ MPA counts on day 4. B and C, show the correlation of net changes (day 4 minus day 3) between CD14++CD16+ percentages/counts and 24 h urinary sodium. Mon2 indicates CD14++CD16+ monocytes; MPA indicates monocyte-platelet aggregates.

Correlation analysis showed that CD14++CD16+ counts were positively associated with CD14++CD16+ MPA counts (**Figure 4**). In addition, net increase in dietary salt intake between day 3 and day 4 was also positively correlated with net increases in CD14++CD16+ percentages and counts (**Figure 4**).

### Monocyte Gene Expression and Intracellular ROS Production

**Figure 5:**
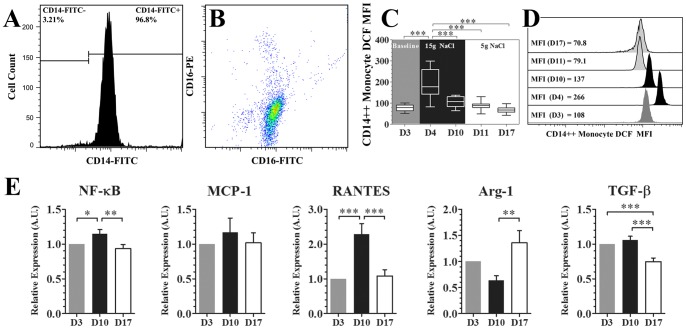
Pro-inflammatory activation of purified CD14++ monocytes during dietary intervention. Figures A to B show the purity analysis of CD14-magnetic-bead isolated human circulating monocytes. A, shows the percentage of CD14++ cells after CD14 bead purification. B, shows a CD14 versus CD16 color plot using cells after CD14 bead purification. Note that monocyte purified by this method are CD14++ monocytes (i.e., CD14++CD16− and CD14++CD16+, according to gating strategies described in **Figure 2**). Figures C and D show intracellular ROS production in magnetic beads purified CD14++ monocytes by 2′, 7′-dichlorofluorescein (DCF) flow cytometry analysis (n = 6, one way repeated measures ANOVA).Figure E shows the related gene expression in magnetic-bead-purified CD14++ monocytes (n = 16, one way repeated measures ANOVA). “D3” to “D17” labeled in the x-axis indicates “day 3″ to “day 17″. *P<0.05, **P<0.01, ***P<0.001. NF-κB indicates nuclear factor kappa B; MCP-1 indicates monocyte chemoattractant protein-1; RANTES indicates Regulated on Activation, Normal T Cell Expressed and Secreted (*or* CC chemokine ligand 5, CCL5); TGF-β indicates transforming growth factor β; Arg1 indicates arginase 1; MFI indicates median fluorescence intensity.

The purity of CD14-bead-isolated monocytes is 90%–98% and is mostly composed of CD14++ cells (CD14++CD16− and CD14++CD16+ subsets, **Figure 5A and 5B**). A significantly enhanced intracellular ROS production in purified CD14++ monocytes was also observed on day 4 (one day after high-salt loading, **Figure 5C and 5D**). As show in **Figure 5E**, nuclear factor kappa B (NF-κB), RANTES (*or* CC chemokine ligand 5, CCL5) and MCP-1 (not reaching statistical significance) mRNA expression were up-regulated during the high-salt phase, which could be reversed by a low-salt diet. In addition, a low-salt diet could down-regulate transforming growth factor β (TGF-β) and up-regulate arginase-1 (Arg-1) levels.

### In Vitro Salt Challenge Experiment

**Figure 6:**
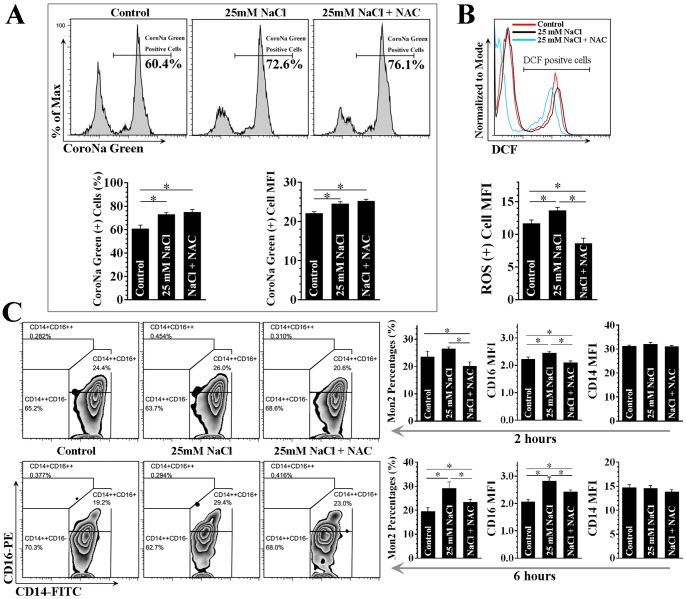
ROS dependent shift towards CD16 positivity in purified human CD14++ monocytes induced by increased extracellular [Na^+^] (25 mM). Figure A shows CD14++ monocyte intracellular [Na^+^] detected by CoroNa Green. B shows intracellular ROS production measured by 2′, 7′-dichlorofluorescein (DCF) flow cytometry in CD14++ monocytes. C shows monocyte subset phenotyping using CD16 and CD14 after CD14++ monocytes were incubated for 2 hours (the upper panel) and 6 hours (the lower panel). N-acetylcysteine (NAC, 2 mM) was used as ROS scavenger. All results are derived from 3 independent tests. MFI indicates median fluorescence intensity; Mon2 indicates CD14++CD16+ monocytes. *P<0.05.

We next tested the hypothesis that elevation in extracellular sodium per se, may lead to expansion of CD14++CD16+ monocytes. Considering there is a developmental relationship between the three monocyte subsets, i.e. from CD14++CD16-, by CD14++CD16+ to CD14+CD16++ monocytes when mobilized from bone marrow [5], [32], CD14 magnetic bead purified circulating monocytes, which are mainly composed of CD14++ monocytes (Figure 5), offers an in vitro model to examine the CD16+ monocyte expansion when challenged with additional sodium in culture medium. As shown in Figure 6A and 6B, adding 25 mM NaCl induced a concomitant increase of intracellular sodium, as well as increased production of ROS, and the latter could be abolished by ROS scavenger. In addition, incubation of CD14++ monocytes with 25 mM for 2 hours, led to CD14++CD16+ cell expansion (Figure 6C, the upper panel). This trend was more obvious when monocytes were incubated for 6 hours (Figure 6C, the lower panel). Moreover, this effect was partially abolished by ROS scavenger. Interestingly, a time-dependent, sodium concentration non-relevant down-regulation of surface CD14 was also observed. These results suggested that increased NaCl per se causes a shift in CD14++ monocytes towards CD16 positivity partially via ROS dependent manner.

### Renal BOLD-MRI and Urinary Markers for Renal Inflammation

**Figure 7:**
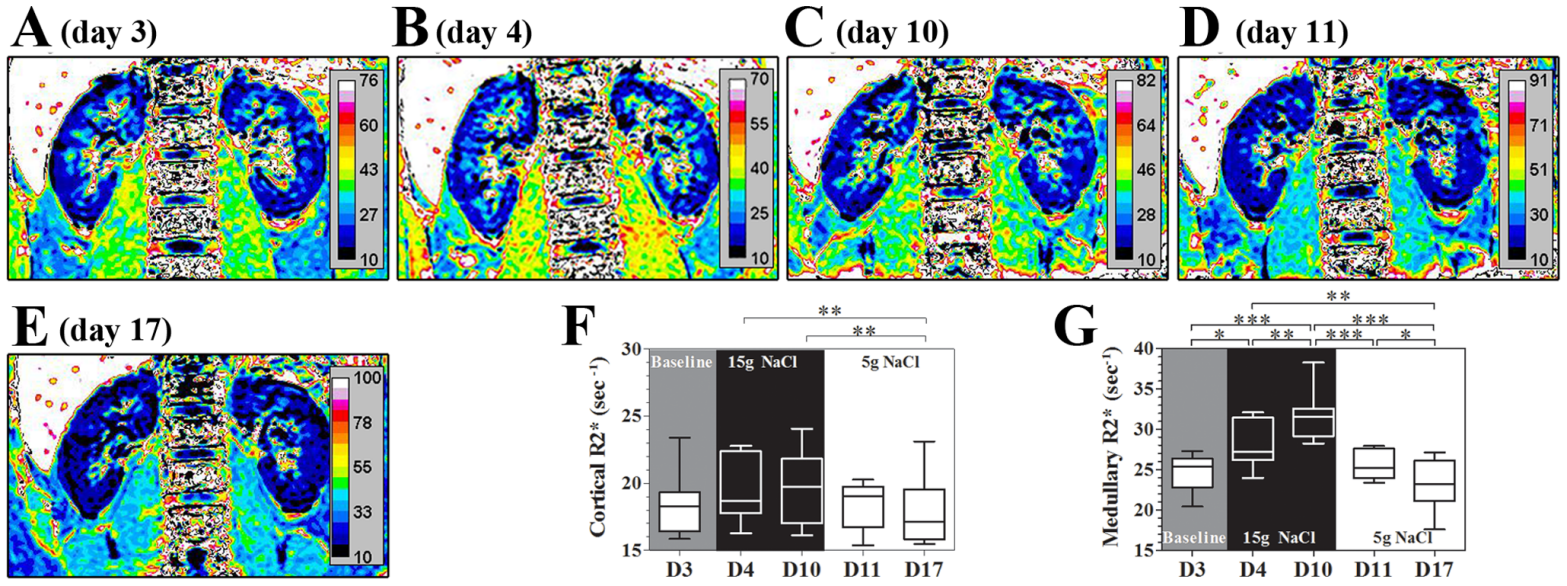
Renal blood oxygen level dependent-magnetic resonance imaging (BOLD-MRI) during dietary intervention. Figures A to E show the representative changes of BOLD-MRI images from one participant during dietary intervention on day 3, day 4, day 10, day 11 and day 17, respectively. Figures F and G show R2*signal changes in renal cortex and medulla, respectively. Statistical comparisons are derived from 11 subjects by one way repeated measures ANOVA. *P<0.05, **P<0.01, ***P<0.001. “D3” to “D17” labeled in the x-axis indicates “day 3″ to “day 17″.

**Figure 8:**
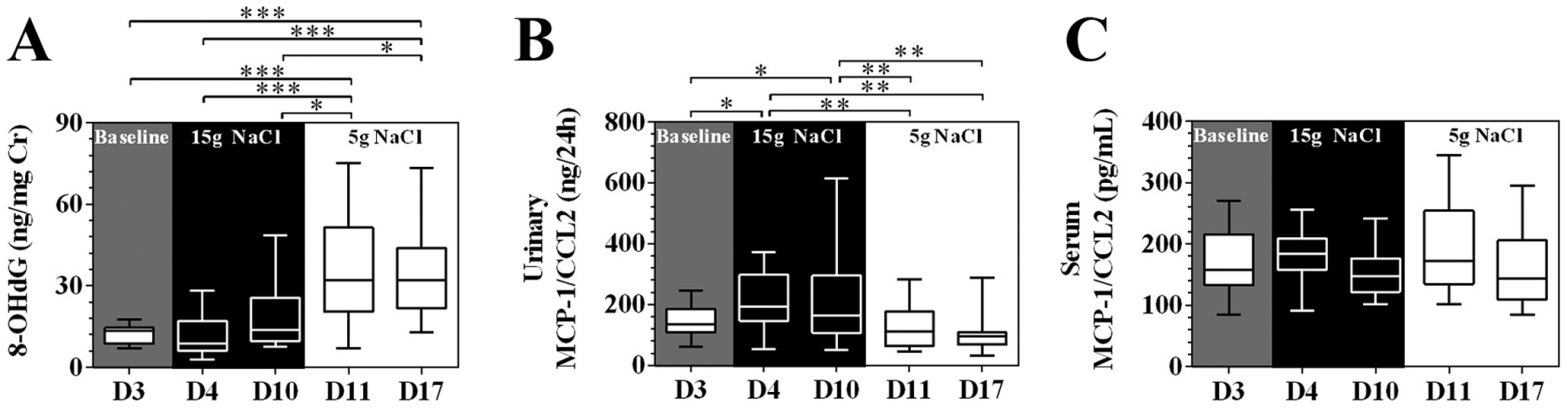
Urinary and serum markers for oxidative stress and renal inflammation. Figure A shows urinary 8-OHdG corrected for urinary creatinine excretion (n = 20, one way repeated ANOVA). Figure B shows 24-h excretion of urinary MCP-1/CCL2 (10 male subjects, one way repeated ANOVA). Figure C shows the dynamic changes of serum MCP-1/CCL2 levels during dietary intervention. No obvious across-time difference was observed in serum MCP-1 levels (one-way repeated measures ANOVA, 10 male subjects). The box and whisker plots: the boxes extend from the 25^th^ to the 75^th^ percentile, with a line at the median. The whiskers extend above and below the box to show the 5^th^–95^th^ percentiles of values. D3 to D17 in x-axis label indicates day 3 to day 17. *P<0.05, **P<0.01, ***P<0.001. 8-OHdG indicates 8-Hydroxy-2-deoxyguanosine; MCP-1 indicates monocyte chemoattractant protein-1.

**Figure 7** demonstrates a remarkable and progressive increase in renal medullary R2* signals (decreased tissue oxygenation) during high-salt loading. Notably, the increased renal medullary R2* signal regressed to its baseline level in a step-down fashion during low-salt feeding. The renal cortical R2* signal remained unchanged during the high-salt phase, whereas at the end of low-salt phase, the cortical R2* signal was reduced compared with the high-salt period. Urinary 8-OHdG, a marker for systemic oxidative stress, [33] was significantly increased on day 11 and reached its plateau at the end of the low-salt phase (**Figure 8A**). Importantly, the dynamic change of urinary MCP-1, but not serum MCP-1 (**Figure 8B and 8C**), followed a similar pattern with those of the CD14++CD16+ subset and renal medullary BOLD-MRI signals (**Figure 7**), indicating a temporal and spatial coincidence of CD14++CD16+ monocytes and renal hypoxia.

## Discussion

In the present work, we demonstrated for the first time that an increase in dietary salt intake could induce a rapid expansion of CD14++CD16+ monocytes, as well as enhance intracellular ROS production by monocytes. This effect decreased during the rest of the high-salt period and was independent of changes in blood pressure levels. In addition, there was no cross-sectional correlation between 24 h urinary sodium and the presence of CD14++CD16+ subset. There was, however, a positive association between the net increase in dietary salt and the net increase in CD14++CD16+ counts/percentages. There results support the interpretation that the magnitude of CD14++CD16+ expansion is more a function of the rapid increase in dietary salt than the absolute level. As anticipated, these alterations could be quickly reversed when transitioned to a low-salt diet.

The plasma sodium concentration is normally maintained within a narrow physiological range by rigorous control systems. However, more and more evidence showed that a transient elevation in plasma sodium concentration and plasma osmolality occur each time especially when high salt diet is consumed [34], [35]. To determine whether or not this elevation directly leads to CD14++CD16+ cell expansion, we adopted an in intro NaCl challenge protocol recommended by Cox and co-workers [36], by adding 25 mM to RPMI 1640 medium (yielding a final [Na^+^] of 159 mM and [Cl^−^] of 132 mM). The results showed that elevation in extracellular [Na^+^] led to a concomitant increase in cytosolic [Na^+^], as well as enhanced ROS production, along with a shift towards CD16 positivity in purified CD14++ monocytes. Importantly, these effects could be partially abolished by ROS scavenger. Taken together with our ex vivo study using purified CD14++ monocytes from subjects during dietary intervention (Figure 5), these data support the interpretation that increased cytosolic [Na^+^] induced mitochondrial ROS production [37], maybe one of the underlying mechanisms contributing to CD14++CD16+ pool expansion observed during high salt intake.

To determine the association between changes in monocyte subsets and end organ inflammation, we focused on renal alterations revealed by BOLD-MRI. This technique offers a non-invasive and reliable tool for assessment of renal hypoxia, which utilizes the magnetic properties of hemoglobin when it converts from the oxygenated to deoxygenated form [38]. Consistent with previous work [30], we found that dietary salt intake influences renal oxygenation (the authors separated salt-loading and depletion with a wash-out period), and we moved forward from this finding by showing that switching from normal salt to high-salt (∼9 g/day to ∼18 g/day in the present work), and transition from high-salt to low-salt (<5 g/day), could induce a change in renal medullary R2* values (inversely correlated with renal tissue oxygenation level). Importantly, dynamics of urinary MCP-1, a well-known marker for renal inflammation and monocyte/macrophage infiltration [18], [39], [40], but not serum MCP-1, presented a similar change to the observed changes in renal oxygenation and circulating monocyte subsets. Taken together, these findings provide human evidence that the rapid expansion of CD14++CD16+ monocytes induced by an abrupt increase in dietary salt intake is associated with renal inflammation.

Admittedly, BOLD-MRI signal is the presentation of “net” tissue oxygen bioavailability, and it cannot differentiate between changes in oxygen delivery, consumption, utilization efficiency and diffusion [41], [42]. In this regard, we speculated from our data that, the initial increase in R2* signal after one day of high-salt loading is the consequences of increased GFR and tubular sodium transport (increased oxygen consumption), and serves as a maintaining factor for renal hypoxia during high-salt loading. Further increases in the R2* signal might be due to the participation of monocyte recruitment and induced oxidative stress (GFR and sodium intake remained constant during high-salt loading), which contributes to reduced mitochondrial oxygen utilization and nitric oxide bioavailability, leading to renal hypoxia [41]. Notably, the feasibility of BOLD-MRI in evaluating organ inflammatory response and oxidative stress has been reported recently [43], [44]. In addition, evidence of systemic oxidative stress during high-salt loading, as shown by increased urinary 8-OHdG, an oxidized nucleoside of DNA and hallmark for oxidative stress, as well as enhanced intracellular monocyte ROS production, strengthens the notion that oxidative stress is closely associated with high-salt loading induced target organ inflammation (elevated urinary 8-OHdG during the low-salt phase indicates a time delay between DNA oxidative damage and DNA base excision repair [33]).

Among the three subsets of monocytes, CD14++CD16+ cells have been increasingly recognized as a major player in inflammation. The results from subpopulation gene expression analyses [23], [45], [46], showed that CD14++CD16+ monocytes express markers superior for endothelial adhesiveness, antigen presentation, angiogenesis and ROS production, indicating their active participation in inflammation. Prior studies also suggest that monocyte CX3CR1 and CCR2 are important for their recruitment, activation and tissue infiltration [34], [47], and target organ injury [35]. Among the three monocyte subsets, CD14++CD16+ monocytes are the only subpopulation that highly express CX3CR1 and CCR2, thus making these cells a potential candidate for preferential recruitment to the kidney after high-salt loading and subsequent inflammation. More recently, the importance of CD14++CD16+ monocytes in cardiovascular disease was reinforced by the finding from Heine’s group that CD14++CD16+ monocytes could independently predict cardiovascular events in a large cohort [11].

In the present study, we also demonstrated a parallel relationship between MPA dynamics and monocyte subset changes in response to a variation in dietary salt intake. The formation of MPAs is largely dependent on binding of P-selectin (CD62P) in activated platelets with a monocyte receptor, P-selectin glycoprotein ligand-1 (PSGL-1), which renders a functional interaction for mutual activation [15], [32]. Recent studies showed that an increase in MPAs is closely associated with thromboembolic events, and MPAs are regarded as a sensitive marker for *in vivo* platelet activation [10], [13], [14], [48], [49]. Notably, CD14++CD16+ monocyte specific MPAs displayed a continuously-increasing trend during high-salt loading, and then underwent an abrupt drop during the low-salt phase. This phenomenon further highlights the unique feature of CD14++CD16+ monocytes in underlying disease processes. Interestingly, a recent work demonstrated a weak but statistical association between aspirin resistance and estimated GFR [50], implying a potential impact of platelet responsiveness on renal function. However, from the data available and our results, it’s still immature to establish a causal role between platelet responsiveness and renal function.

The present study has the following limitations. First, although a temporal and spatial correlation was clearly demonstrated, the observational nature of this work did not allow verification of a causal linkage between monocyte subset hemostasis and renal function in response to fluctuation in dietary salt intake. Second, the currently available magnetic bead-based method for isolating monocyte subsets is relatively complicated and is not suitable for a population based study. Consequently, we only isolated and purified CD14++ monocytes, and the interpretation of gene expression changes could not be applied to a certain subset. Third, the choice of anticoagulant agent would have an impact on platelet-related assays, especially on monocyte subsets due to their similarities. In addition to sodium citrate used in this work and by other groups [24], EDTA is also widely used for monocyte subset and MPA analysis. Thus, standardization of anticoagulant agents is warranted for future cross-study comparisons.

An interesting work by Titze’s group has proposed a protective role of macrophage infiltration and activation in skin during high-salt loading, via vascular endothelial growth factor C-dependent lymphangiogenesis to buffer high-salt induced volume load [51]. The relative contribution of monocyte/macrophage infiltration in target organs versus “off-target” organs in response to high-salt loading remains to be established.

In conclusion, for the first time, we demonstrated a rapid expansion of the CD14++CD16+ monocyte pool and platelet activation, as well as monocyte pro-inflammatory activation in response to increased dietary salt intake. In addition, monocyte recruitment and infiltration, presumably CD14++CD16+ monocytes, is associated with high-salt intake induced renal hypoxia. Our findings reveal novel pathophysiological links between dietary salt intake, innate immunity and target organ inflammation. Future work is warranted to elucidate whether CD14++CD16+ monocytes may serve as a potential target for new therapeutic strategies in high-salt intake induced end organ injuries.
